# Serum Extracellular Vesicle Protein Signatures Associated with Early-Stage High-Grade Serous Ovarian Carcinoma

**DOI:** 10.3390/cells15080706

**Published:** 2026-04-16

**Authors:** Michelle Lightfoot, Kalpana Deepa Priya Dorayappan, Vignesh Vudatha, Lakshmi Narasimhan. Chakrapani, Priyam Das, Lianbo Yu, Colin Hisey, Takahiko Sakaue, Thangavel Muthusamy, Parthiban Panneerselvam, Floor Backes, Casey Cosgrove, Derek Hansford, David E. Cohn, David M. O’Malley, Rajan Gogna, Karuppaiyah Selvendiran

**Affiliations:** 1 Division of Gynecologic Oncology, Department of Obstetrics and Gynecology, NYU Langone Health/Perlmutter Cancer Center, New York, NY 10016, USA; michelle.lightfoot@nyulangone.org; 2 Division of Gynecologic Oncology, Department of Obstetrics and Gynecology, The Ohio State University Wexner Medical Center, Columbus, OH 43210, USA; kalpanadeepapriya.dorayappan@osumc.edu (K.D.P.D.); lakshmi.chakrapani@osumc.edu (L.N.C.);; 3 Department of Surgery, Virginia Commonwealth University School of Medicine, 1200 E Broad St., Richmond, VA 23298, USA; vignesh.vudatha@vcu.edu; 4 Department of Biostatistics, Virginia Commonwealth University, Richmond, VA 23298, USA; priyam.das.1@vcu.edu; 5 Department of Biomedical Informatics, The Ohio State University Wexner Medical Center, Columbus, OH 43210, USA; linabo.yu@osu.edu; 6 Department of Biomedical Engineering, The Ohio State University, Columbus, OH 43210, USA; 7 Division of Gastroenterology, Department of Medicine, Kurume University School of Medicine, 67 Asahi-machi, Kurume 830-0011, Japan; 8 Cellular and Molecular Biochemistry, Sree Balaji Medical College and Hospital, Bharath Institute of Higher Education and Research, Chennai 600073, Tamil Nadu, India; 9 University of The Cumberlands, Williamsburg, KY 40769, USA

**Keywords:** high-grade serous ovarian carcinoma, extracellular vesicles, biomarkers, serum EV proteins, ovarian cancer

## Abstract

**Highlights:**

**What are the main findings?**

- This study identifies extracellular vesicles (EVs) as a promising source of protein biomarkers for the early detection of HGSOC.
- EV-associated proteins demonstrate greater sensitivity and specificity than traditional serum proteins.

**What is the implication of the main findings?**

- These findings contribute to advancements in discovering and validating novel EV proteins as biomarkers for early-stage HGSOC.

**Abstract:**

**Background:** High-grade serous ovarian carcinoma (HGSOC) is the most common and lethal subtype of epithelial ovarian cancer and is frequently diagnosed at advanced stages. Because currently available blood-based biomarkers have limited performance in early-stage disease, there is a need to identify circulating biomarker candidates associated with early-stage HGSOC. In this retrospective multi-institutional case–control study, we evaluated whether serum extracellular vesicle (EV)-associated protein signatures distinguish early-stage HGSOC from healthy controls. **Methods:** Serum samples (*n* = 252) were obtained retrospectively from multiple institutions and included healthy controls and patients with early- and advanced-stage HGSOC. EV-associated proteins were profiled using liquid chromatography–tandem mass spectrometry (LC–MS/MS) and proximity extension assay (PEA) to identify candidate proteins enriched in early-stage HGSOC. Selected candidates were evaluated by enzyme-linked immunosorbent assay (ELISA), and tissue-level expression was examined in early-stage HGSOC specimens. A multimarker combination model was generated using a smoothed empirical estimate of hyper-volume under the manifold (SHUM) approach and internally assessed by leave-one-out cross-validation. **Results:** Ten EV-associated serum proteins were prioritized on the basis of differential expression and fold change and were confirmed to be expressed in early-stage HGSOC tissues. In ELISA-based analyses, the combined 10-protein EV panel distinguished early-stage HGSOC from healthy controls with an area under the curve (AUC) of 0.99 in the study dataset, whereas MUC16 (CA-125) showed substantially lower performance in this comparison. The SHUM-based model yielded a true-positive rate of 0.971, a false-positive rate of 0.057, and a Matthews correlation coefficient of 0.915 in the analyzed cohort. Several candidate proteins were differentially enriched in EV fractions but not in matched whole serum. **Conclusions:** Serum EV-associated proteins are altered in early-stage HGSOC and define a multi-protein signature associated with this disease state in a retrospective case–control setting. These findings support further evaluation of EV-based biomarker candidates in clinically representative and prospectively collected cohorts that include benign gynecologic conditions, symptomatic patients, and pre-diagnostic samples.

## 1. Introduction

Epithelial ovarian cancer is an aggressive malignancy with a high fatality rate, and most patients present with advanced-stage disease [1,2,3]. High-grade serous ovarian carcinoma (HGSOC) is the most common histologic subtype and accounts for the majority of ovarian cancer-related deaths. Survival is strongly associated with stage at diagnosis, with substantially better outcomes observed in patients diagnosed with stage I–II disease than in those diagnosed with stage III–IV disease. These observations have motivated continued efforts to identify circulating biomarkers associated with earlier-stage HGSOC [4,5,6,7].

Although biomarkers are attractive candidates for ovarian cancer detection and disease stratification, the currently available serum markers have important limitations [8,9,10,11,12,13]. CA-125 remains the most widely used biomarker in ovarian cancer, but its sensitivity and specificity are inadequate for reliably identifying early-stage HGSOC, and its performance may be affected by nonmalignant gynecologic and inflammatory conditions [14,15,16]. In addition, many potentially informative tumor-associated proteins are present at low abundance in whole serum, making them difficult to detect reproducibly [17,18].

Extracellular vesicles (EVs) are membrane-bound particles released by cells into body fluids and represent a potentially enriched source of disease-associated biomolecules [19,20,21]. Because EV cargo may reflect the molecular state of the tissue of origin and is relatively stable in circulation, EV-associated proteins have emerged as promising biomarker candidates in cancer [20,22,23]. Beyond their biomarker potential, EVs are also implicated in tumor progression, immune modulation, and metastatic dissemination [24,25,26].

In the present retrospective, multi-institutional case–control study, we sought to identify serum EV-associated proteins that differ between women with early-stage HGSOC and healthy controls, and to compare these profiles with those observed in advanced-stage HGSOC. Our central premise was that early-stage HGSOC is associated with a distinct circulating EV protein profile that can be detected analytically in serum-derived EV fractions. We therefore focused on biomarker discovery and internal evaluation of candidate EV-associated proteins rather than on establishing clinical screening or diagnostic performance. These findings are intended to nominate EV protein candidates for future validation in clinically representative diagnostic and longitudinal screening settings.

## 2. Methods

Human Sample Procurement and Processing: Serum samples were retrospectively obtained from The Ohio State University James Cancer Hospital and Solove Research Institute, Inova Schar Cancer Institute, Cedars-Sinai Samuel Oschin Cancer Institute, and Washington University Siteman Cancer Center under institutional review board approval (IRB study number 2021C0031). Specimens were obtained from the respective biobanks at each institution after consent was given for specimen collection. Separate consent was not required from participants in order to obtain the de-identified clinical samples. De-identified clinical data included age, diagnosis, histology, grade, disease stage, and treatment status at the time of collection. HGSOC samples were included only if collected before systemic chemotherapy. Patients with borderline tumors or low-grade carcinomas were excluded. Control samples were included only if there was no documented adnexal pathology, malignancy, or major comorbid condition at the time of collection.

A total of 252 serum samples were analyzed, including 75 controls, 74 early-stage (I/II) HGSOC cases, and 87 advanced-stage (III/IV) HGSOC cases. Samples were contributed by Ohio State University (n = 138), Inova Schar Cancer Institute (n = 76), Cedars-Sinai Samuel Oschin Cancer Institute (n = 16), and Washington University Siteman Cancer Center (n = 14). The median patient age was 60.4 years (range, 39–81 years). Sample distribution is summarized in Supplemental Table S1.

All serum specimens were stored at −80 °C until analysis. For EV isolation experiments, samples were thawed on ice and processed under standardized laboratory conditions. To minimize pre-analytical variation, repeat freeze–thaw cycles were avoided whenever possible, and comparable downstream workflows were applied across groups. However, because this was a retrospective multi-institutional study, detailed information regarding blood draw-to-processing intervals, storage duration, and all site-specific pre-analytical handling variables was not uniformly available for every specimen. Accordingly, potential pre-analytical and site-related variability should be considered when interpreting the findings. These limitations are acknowledged in the Discussion Section.

Extracellular vesicles(EV) isolation by microfluidic device (MFD): The process of EV isolation and confirmation by MFD is shown in Supplemental Figure S1A,B. The serum samples were thawed on ice and diluted 1:5 in 0.22 µm filtered PBS. The diluted serum was processed through the MFD, which was coated with the EV-specific surface marker antibodies CD9, CD63, and TSG101 and epithelial surface marker EpCAM. The devices were then washed with 0.22 µm filtered PBS at a 25-to-30 μL/min flow rate for 10 min. EVs captured on the device were eluted with 400 μL of Glycine–HCl buffer at 80 μL/min, followed by pressurized air through channels to ensure complete collection, as we previously described [27,28,29]. The collected EVs were stored at −80 °C until further downstream processing, as previously described [27,28]. Validation of EV isolation and purification by MFD was performed by testing across Dr. Karuppaiyah’s and Dr. Hansford’s laboratories and compared with other standard methods, including ultracentrifugation and the commercial Exoquick kit (System Biosciences, CA, USA). 

EV isolation by ultracentrifugation: EVs were isolated from 250 μL of cell-free serum. Samples were first thawed on ice and then diluted in 1 mL 1× phosphate-buffered saline (PBS). The diluted serum samples were centrifuged at 400× *g* for 15 min to remove debris, and the supernatants were transferred to a fresh tube and centrifuged at 10,000× *g* for 30 min to remove large-sized microvesicles and apoptotic vesicles. The supernatants were then filtered through 0.22 μm porous membranes followed by a spin at 100,000× *g* for 90 min at 4 °C to isolate the EVs. The EV pellets were then washed in 1 mL of 1 × PBS followed by a second step of ultracentrifugation at 100,000× *g* at 4 °C for 90 min. The final pellet was re-suspended in PBS, and the vesicular protein concentration was determined using the direct detect (R) spectrophotometer.

EV isolation by commercial kit: The EVs were isolated from HGSOC or control patient serum samples (100 µL) using the Exoquick kit solution according to the manufacturer’s (System Biosciences) protocol. The resulting EV pellet was re-suspended in either 100 µL of cold PBS or lysis buffer, depending on the downstream application. Because the isolation approaches used in this study enrich a heterogeneous population of small extracellular vesicles, we use the term “extracellular vesicles (EVs)” throughout the manuscript rather than using “EVs” interchangeably [29].

EV confirmation and quantification—Nanoparticle tracking analysis (NTA): After the capture and release of the vesicles from the microfluidic device, serum EV samples were diluted with PBS (1:4) and (1:10). For reliable NTA measurement, we used an appropriate EV sample dilution that provides approximately 20–100 particles in the field of view [30]. NTA post-acquisition settings were optimized and kept constant between samples. Each video was then analyzed to give the mean, mode, and median particle size and an estimated number of particles per mL serum. We recorded three 10 s videos on the NanoSight LM10, which were analyzed using tracking software. The NTA software plots particle size range versus particle number in the sample, and 100 nm polystyrene latex microspheres were routinely analyzed to confirm instrument performance [28].

ImageStream Flow Cytometry (ISF): The ImageStream was equipped with a 60× objective to detect particles in the EV/extracellular vesicle size range, with a Numerical Aperture (NA) of 0.9 and a resolution of 3 μm^2^/pixel. Although many of the vesicles are below the size of the pixel resolution when labeled with fluorescent molecules, these smaller vesicles become detectable due to the intensity of the signal, and the width of the core stream is reduced to 7 μm to increase the frequency of in-focus objects. In addition, the speed was set to the lowest setting for maximum resolution. All sheath buffers were filtered with 0.1 μm filters to ensure minimal background particulates. The instrument was equipped with 488 nm (blue), 561 nm (green), and 642 nm (red) lasers to be compatible with the Exo-FITC, PE, and AF647 fluorophores [27]. Signal intensity was set to 200 mW to increase the number of photons generated per fluorochrome molecule, including the 758 nm laser for scatter measurements [27].

EV size and morphology were then assessed using scanning electron microscopy (SEM) and Classical Transmission electron microscopy (TEM). EV morphology was analyzed by classical TEM using negative staining. Purified EVs were fixed in 0.1% glutaraldehyde, applied to glow-discharged Formvar/carbon-coated grids, and incubated for 20 minutes. Grids were washed with PBS, post-fixed in 1% glutaraldehyde, rinsed with distilled water, and stained with uranyl acetate for 1 minute. SEM: Channels were disassembled and treated with Karnovsky’s fixative (2.5% EM-grade glutaraldehyde, 2% formaldehyde, and 0.1 M sodium phosphate buffer) for 10 min and washed with PBS twice for 5 min, followed by a series of dehydration treatments of 35% ethanol for 10 min, twice with 50% ethanol for 10 min, twice with 70% ethanol for 10 min, twice with 90% ethanol for 10 min, and four times with 100% ethanol for 10 min. Samples were then coated with palladium/gold and imaged with an InLens detector at 3 keV and 100% charge compensation (Zeiss Ultra 55 SEM).

TEM observation was performed using low-dose mode on an FEI Tecnai F20 TEM according to a previously published protocol by Gao et al. [31].

Evaluation of EV proteome profiles in high-grade serous ovarian cancer compared to controls—LC-MS/MS: Protein identification was performed using nano-liquid chromatography–nanospray tandem mass spectrometry (LC-MS/MS) on a Bruker tims-ToF Pro equipped with a Captive Spray source operated in positive ion mode. Samples were separated on a C18 reverse-phase column (1.6 µm, 250 mm × 75 µm IonOpticks) using a Bruker nano Elute UHPLC system. A total of 200 ng of peptide was injected for each analysis. Pre-injection, the column was equilibrated with 4 column volumes at 800 bar. Mobile phase A was 0.1% formic acid in water, and acetonitrile (with 0.1% formic acid) was used as mobile phase B. The flow rate was set to 0.4 µL/min. Mobile phase B was increased from 2 to 17% over the first 60 min, then increased to 25% over the next 10 min, further increased to 37% over the next 10 min, and finally increased to 80% over 10 min and then held at 80% for 10 min. MS and MS/MS experiments were recorded over the m/z range of 100–1700 and K0 of 0.6–1.6. PASEF was used for all experiments, with the number of PASEF MS/MS scans set to 10. Active exclusion was applied and released after 0.4 min, with precursor reconsidered if the current intensity/previous intensity was 4.0 or greater.

EVs protein quantification and analysis—ELISA: EVs were lysed using RIPA buffer added with protease cocktail inhibitors and incubated at room temperature for 5 min. Protein quantification was performed using the direct detect infrared spectrophotometer or NanoDrop UV-Vis spectrophotometer. The plates were coated with equal concentrations of EV proteins and incubated on a 96-well assay plate for 48 h and then probed with the optimized primary- and secondary-antibody concentration titrated for each protein target. With the addition of the TMB substrate solution to the HRP-conjugated secondary antibody, a blue color develops, which corresponds to the target protein concentration. The reaction was then stopped by the addition of 0.2N Sulphuric acid, which turns the reaction mixture to a yellow color. Readings were obtained within 10 min at 405 nm.

Database Searching: RAW Bruker.d mass spectrometry files from all samples (n = 12) were converted to mzML with ProteoWizard [30] and OpenMS (v 2.5.0) [31] with an in-house Nextflow script. Converted files were searched on the OpenMS platform with the MSGF+ search engine against a reviewed UniProt human proteome (downloaded 24 September 2020) containing the cRAP and MaxQuant contaminant FASTAs. Search parameters included full trypsin digest, one missed cleavage, carbamidomethylation of cysteine as a fixed modification, and oxidation of methionine as a variable modification with precursor and fragment mass tolerances of 20 ppm and 0.05 Da. PSM rescoring was performed with Percolator, and protein inference was performed with Epifany across all samples, setting peptide and protein false-discovery rates to 0.05.

Statistical Analysis: Protein counts were normalized by the TMM method. The R package limma was used to compare the normalized counts of early-stage HGSOC versus control groups by fitting through an empirical Bayes method with the VOOM method to estimate the mean–variance trend. Differentially expressed proteins were selected using both fold change and significance cutoff by controlling for the expected mean number of false positives. For the ELISA, proteins were analyzed based on statistically significant differences in the average expression by groups, and the confirmed proteins were assessed for the detection of early-stage ovarian cancer in humans from healthy controls using ROC curves. Sensitivity and specificity were calculated, and ROC curves and AUC values were generated using R package pROC (version 1.19.0.1).

Multiple approaches exist for linearly combining multiple biomarkers, with a focus on optimizing the area under the ROC curve (AUC) for disease subgroup classification, as emphasized by Pepe and Thompson [32]. In the context of multicategorical ordered outcomes, AUC is referred to as Hyper-volume Under Manifolds (HUM). The search for the most effective combination of biomarkers for classification often involves maximizing HUM. However, as HUM cannot be directly measured, it is common practice to maximize the empirical estimate of HUM (EHUM) to identify the optimal combination vector. Maiti et al. [33] introduced a refined estimate of HUM, termed the smoothed empirical estimate of HUM (SHUM), and proposed maximizing SHUM instead of directly optimizing EHUM to determine the optimal combination vector. To ensure the identifiability of the combination vector, we constrain the Euclidean norm of the biomarker coefficients to be 1. We employ the optimization algorithm proposed in Das et al. [34] to maximize the SHUM criterion under this constraint.

In our ELISA dataset, we have collected measurements from 35 early-stage HGSOC patients and 35 controls. The SHUM method was validated by using the leave-one-out (LOO) validation technique to validate the out-of-sample predictive performance of the SHUM method. In this approach, we train the model on 69 subjects and then test it on the remaining subject. This process is repeated 70 times, with each subject being excluded once. To make predictions for the left-out subject, we compute a score based on the optimal biomarker combination. If the score surpasses a threshold value, we predict the patient as positive for HGSOC; otherwise, we classify the patient as a control. The threshold value for each repetition is determined using Youden’s index [35], a metric assessing the diagnostic accuracy of a test.

## 3. Results

### 3.1. Identification of EV-Associated Proteins Linked to Early-Stage HGSOC

**Figure 1:**
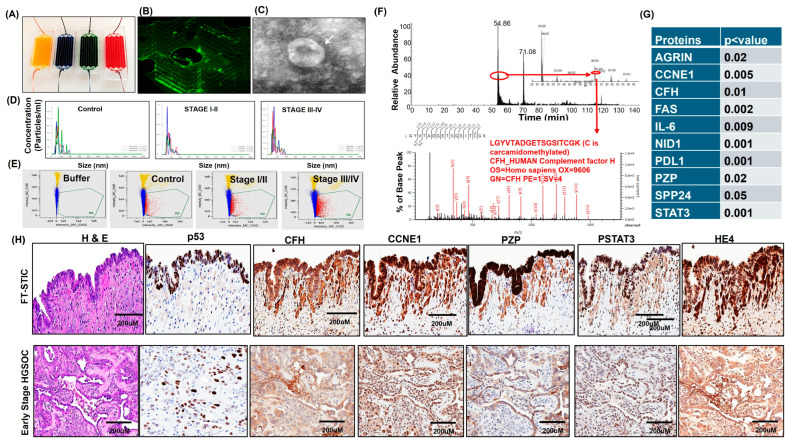
**Isolation, characterization, and protein identification of extracellular vesicles (EVs).** (**A**) Microfluidic device (MFD) functionalization and exosome capture image of the microfluidic device (MFD) with channels functionalized using CD9 or CD63 antibodies, represented by different flow colors. (**B**) Successful capture of Exo-FITC labeled exosomes (green) within MFD channels. (**C**) Morphological characterization and size measurement of EVs (indicated by white arrows) using Classical Transmission Electron Microscopy (TEM). (**D**) EV particle concentration and size validation. Quantification and sizing of EVs were confirmed through nanoparticle tracking analysis (NTA) in control samples, as well as in serum samples from HGSOC patients at stage I/II and stage IV. (**E**) Validation of EV secretion particles using image stream flow cytometry, with the surrounding color bars indicating exosome counts, in control samples, benign samples, and HGSOC serum samples at stage I/II and stage IV. (**F**) Isolation of serum EV proteins from controls, early-stage, and advanced-stage HGSOC samples using the MFD chip or ExoQuick kit. Subsequently, these proteins were subjected to LC-MS/MS or shotgun proteomics (n = 6), arrow marked the identified candidate protein peak. (**G**) Selection of EV candidate proteins: Candidate proteins from EVs were chosen for confirmation based on fold change and statistical significance. (**H**) Confirmation of candidate proteins in STIC and early-stage HGOSC patient tissues using IHC (n = 5).

Extracellular vesicles (EVs) were isolated from serum samples obtained from healthy controls and from patients with early (stage I/II) or advanced (stage III/IV) HGSOC using either our microfluidic device (MFD) or the ExoQuick precipitation method (Figure 1A). FITC-labeled EVs captured within MFD channels demonstrated efficient enrichment of EV populations (Figure 1B). EV morphology and size distribution were confirmed by cryo-TEM and SEM, which revealed intact spherical vesicles consistent with EVs (Figure 1C; Supplementary Figure S1A). Imaging flow cytometry further confirmed expression of canonical EV-associated markers, including CD9 and CD63, across control- and HGSOC-derived EV preparations (Supplementary Figure S1B–C). NTA and imaging-based quantification showed that the MFD platform yielded adequate EV concentrations from control, early-stage, and advanced-stage HGSOC serum samples (Figure 1D,E; Supplementary Figures S2 and S3A,B). EV concentrations were higher in advanced-stage HGSOC than in early-stage disease and controls, whereas EV size distributions were broadly comparable across groups.

To identify candidate proteins associated with early-stage HGSOC, we performed LC-MS/MS-based proteomic profiling of serum EVs from early-stage HGSOC cases and healthy controls (Figure 1F). Multiple proteins were enriched in EVs from early-stage HGSOC, and differential expression results are summarized in Figure 1G. Pathway analysis of the discovery set suggested enrichment of networks involved in cell proliferation, survival, tumor progression, and metastasis (Supplementary Figure S4A,B; Supplementary Table S2). Follow-up analyses confirmed differential expression of prioritized EV-associated proteins among controls, early-stage HGSOC, and advanced-stage HGSOC. Several candidate proteins were also detected in serous tubal intraepithelial carcinoma (STIC) lesions and in early-stage HGSOC tissues (Figure 1H; Supplementary Figure S5), supporting biological relevance to HGSOC-associated disease processes. Collectively, these findings identify a set of circulating EV-associated proteins linked to early-stage HGSOC in this retrospective cohort.

### 3.2. Combined EV-Associated Proteins Distinguish Early-Stage HGSOC from Healthy Controls in the Study Cohort

**Figure 2:**
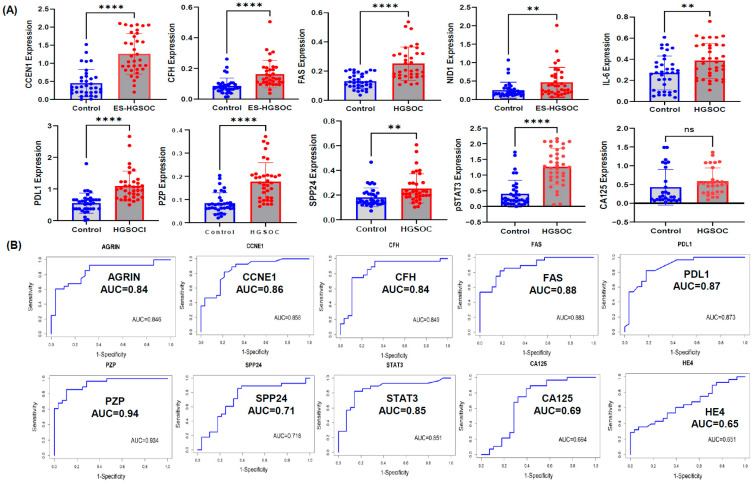
**Serum EV protein training cohort study:** (**A**) Serum EV was isolated from early-stage HGSOC samples using MFD and quantified by imaging flow cytometry (ISF). Serum EV candidate proteins were analyzed by ELISA in control and early-stage (ES) HGSOC samples (n = 41; ** *p* < 0.005; **** *p* < 0.0001; ns is non significant). CA125 expression was analyzed by ELISA in control and ES-HGSOC samples (n = 27). (**B**) Identifying EV candidate protein sensitivity and specificity: Area under curve (AUC) of AGRIN, CFH, PZP, CCNE1, FAS, SPP24, STAT3, PDL-1, IL-6 and NID1 based on ELISA results from serum EV isolated from control and early-stage serum samples; all our candidate EV proteins had an AUC of greater than 0.84 (n = 30). CA125 (aka MUC16) and HE4expressions were analyzed by ELISA in a new set of early-stage patient serum samples; serum CA125 and HE4 had an AUC close to 0.65 and 0.69 respectively (n = 27).

To assess whether combinations of EV-associated proteins improved analytic discrimination between groups, we evaluated 10 candidate biomarkers selected on the basis of fold change and statistical significance: ARGIN, CCNE1, CFH, FAS, IL-6, NID1, PD-L1, PZP, SPP24, and STAT3. These proteins were quantified by ELISA in an independent study subset comprising 35 early-stage HGSOC cases and 35 age-matched healthy controls (Figure 2A). All 10 candidates were significantly elevated in early-stage HGSOC relative to controls and demonstrated stronger performance in EV fractions than in whole serum (Figure 2B; Supplementary Figures S6 and S7). As shown in Figure 2, several individual EV-associated proteins demonstrated strong discriminatory performance, with AUC values reaching approximately 0.94, whereas the optimally combined multimarker model achieved an AUC of 0.99 in the study dataset.

MUC16 (CA-125), assessed in this dataset by ELISA, showed limited ability to distinguish early-stage HGSOC from healthy controls (AUC = 0.427; *p* = 0.143; Supplementary Figure S8). Accordingly, MUC16 was not included in the final combined EV biomarker model. By contrast, MUC16 measured in whole serum showed substantially lower discriminatory performance in this comparison.

**Figure 3:**
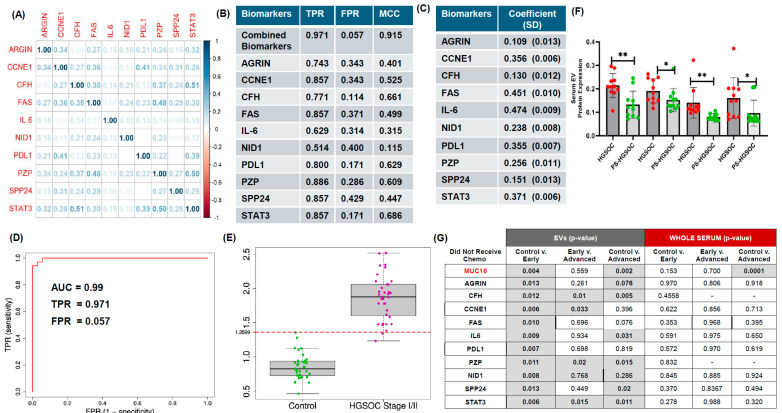
**EV candidate proteins predict presence of early-stage high-grade serous carcinoma.** (**A**) Correlation plot of considered biomarkers (ARGIN, CCNE1, CFH, FAS, IL-6, NID1, PDL1, PZP, SPP24, STAT3) across all patients. (**B**) Out-of-sample prediction performance by optimal combination of biomarkers, obtained via maximizing smoothed empirical estimate of HUM (SHUM), and individual biomarkers are noted down. For each candidate biomarker, leave-one-out (LOO) out-of-sample true-positive rate (TPR), false-positive rate (FPR) and Mathew’s correlation coefficient (MCC) are reported for ELISA dataset. (**C**) Optimal coefficients of biomarkers in the optimal biomarker combination for maximizing diagnostic accuracy of early-stage HGSOC are noted. Bootstrap standard errors of the coefficients are calculated based on 500 bootstrap samples and are given within parentheses. (**D**) Receiver operating characteristic (ROC) curve for diagnosing early-stage HGSOC via optimally combined biomarker. Confirmation of candidate proteins using ELISA in early-stage HGSOC patient samples compared to control samples (without cancer). AUC = 0.99; TPR = 0.943; FPR = 0. Combined biomarker: 0.109 * AGRIN +0.356 * CCNE1 +0.13 * CFH + 0.451 * FAS + 0.471 * IL6 + 0.28 * NID1 + 0.355 * PDL1 + 0.256 * PZP + 0.151 * SPP24 + 0.361 * STAT3. Optimal cut point = 1.35991 (HGSOC +ve if score is greater than cutoff). (**E**) Boxplots of the optimal combination vector scores are shown across the outcome categories for all patients. (**F**). Serum EV and protein profiles before and after HGSOC surgery. Serum EV candidate proteins were analyzed by ELISA in HGSOC and PS-HGSOC samples (n = 11; * *p* < 0.01; ** *p* < 0.005). (**G**) Candidate protein expression in EVs and whole serum across controls, early- and advanced-stage HGSOC by ELISA (n = 12).

Because the individual biomarkers showed only modest pairwise correlations (Figure 3A), we next assessed whether a linear combination of these EV-associated proteins improved classification performance. We used a SHUM-based optimization framework to derive an optimal combination vector under a unit-norm constraint on model coefficients. In internal analyses, the combined biomarker model outperformed each individual marker, achieving a true-positive rate of 0.971, a false-positive rate of 0.057, and a Matthews correlation coefficient of 0.915 in the study cohort (Figure 3B).

To evaluate internal out-of-sample performance, we applied leave-one-out cross-validation across the 70-subject ELISA dataset. The resulting combined biomarker score achieved an AUC of 0.99, at the Youden-derived threshold index 1.36 (Figure 3D,E). These findings suggest that combining EV-associated protein markers improves analytic discrimination between early-stage HGSOC and healthy controls in this retrospective dataset. However, because model derivation and assessment were performed within a limited cohort, these results should be interpreted as internally validated discovery findings requiring external confirmation.

### 3.3. Candidate Protein Expression in EVs and Whole Serum

To explore disease association further, we compared candidate protein expression across control, early-stage HGSOC, and advanced-stage HGSOC samples. Several EV-associated proteins, including CFH, CCNE1, FAS, HE4, IL-6, MUC16, pSTAT3, PZP, and STAT3, were elevated in EV fractions from early-stage HGSOC relative to controls (Figure 3G). In contrast, these differences were generally not observed in matched whole-serum analyses. Differences between early-stage and advanced-stage HGSOC EVs were observed for CFH, CCNE1, PZP, STAT3, and VEGF, again highlighting stage-associated heterogeneity within the EV compartment. In whole serum, MUC16/CA-125 was the only analyte that differed significantly among groups, and this was primarily observed between controls and advanced-stage HGSOC. In a limited subset of paired samples, CFH, NID1, PZP, and pSTAT3 showed changes after surgery (Figure 3F), consistent with an association with tumor burden. Given the small size of this paired subset, these observations should be considered preliminary.

## 4. Discussion

High-grade serous ovarian carcinoma (HGSOC) remains the most lethal gynecologic malignancy, and most cases are diagnosed after spread has already occurred [36,37,38]. This persistent clinical challenge has motivated extensive efforts to identify circulating biomarkers associated with earlier-stage disease. In the present study, we identified a panel of serum EV-associated proteins that distinguished early-stage HGSOC from healthy controls in a retrospective multi-institutional case–control cohort and that, when combined, outperformed whole-serum MUC16 in this dataset.

Importantly, the findings should be interpreted within the context of the study design. This is a retrospective case–control biomarker discovery study that compared patients with established HGSOC to healthy controls rather than a clinically representative diagnostic cohort or a prospective screening population. Accordingly, the present work demonstrates strong analytic discrimination within the study dataset, but it does not establish clinical screening performance, pre-diagnostic detection, or real-world diagnostic utility in women presenting with pelvic masses, inflammatory conditions, endometriosis, or other benign gynecologic disorders. We have therefore intentionally reframed our conclusions to emphasize biomarker association and discovery rather than definitive early-detection or diagnostic performance.

This distinction is particularly important in light of the prior ovarian cancer biomarker literature. Several biomarker panels have shown excellent performance in retrospective case–control studies yet failed to outperform CA-125 in pre-diagnostic or clinically representative validation cohorts [20,39]. Similar challenges have been observed in ovarian cancer and other malignancies, where apparently high-performing biomarker signatures derived from diagnostic samples did not generalize when tested in longitudinal collections or in populations with relevant benign and symptomatic comparator groups [39]. Our results should therefore be viewed as hypothesis-generating and as a foundation for future validation, rather than as evidence of immediate clinical applicability.

A second important consideration is the biological heterogeneity of HGSOC. Although early-stage disease is associated with better survival, HGSOC does not necessarily progress through a uniform, prolonged, and linear stage I–II trajectory in all patients [6]. Distinct tumors may differ substantially in dissemination kinetics, metastatic competence, immune interactions, and intrinsic aggressiveness [40,41]. Thus, the EV-associated proteins identified here may reflect, at least in part, specific biological phenotypes of HGSOC that are overrepresented among tumors diagnosed at an earlier stage, rather than universal temporal markers of all HGSOCs prior to progression. This possibility is consistent with the observation that some EV proteins also distinguished early-stage from advanced-stage disease, suggesting that the identified signature may capture disease state or phenotype-associated biology in addition to stage-related differences.

Despite these caveats, several findings support the biological relevance of the EV candidates identified here. Multiple proteins in the panel have known roles in ovarian cancer or cancer-associated signaling, including CCNE1, STAT3, IL-6, and PD-L1. CCNE1 is of particular interest because its amplification and overexpression are implicated in genomic instability, transformation of fallopian tube secretory epithelial cells, platinum resistance, and poor prognosis in HGSOC [27,42,43,44]. STAT3 likewise has a well-established role in ovarian tumor progression and immune modulation [30,42]. Other candidates, including CFH, NID1, PZP, and SPP24, are less studied in HGSOC but may reflect tumor–microenvironment interactions, immune evasion, or extracellular matrix remodeling [45,46]. The observation that several markers were enriched in EV fractions but not in matched whole serum supports the concept that the EV compartment may concentrate biologically informative tumor-associated signals that are diluted or obscured in unfractionated circulation.

The present study also has several methodological limitations. First, although samples were processed under standardized experimental workflows after receipt, this was a retrospective multi-institutional collection, and comprehensive pre-analytical metadata were not uniformly available for all specimens. Variables such as blood collection-to-processing interval, long-term storage duration, and site-specific handling may have contributed to variability in EV yield or proteomic measurements. Second, the comparator group consisted of healthy controls rather than women with benign gynecologic disease, adnexal masses, inflammatory conditions, or non-gynecologic illnesses, which likely inflates apparent specificity relative to clinically relevant diagnostic settings. Third, the combined biomarker model was derived and internally assessed within a relatively small dataset, with performance estimated using leave-one-out cross-validation. Although this provides internal support, it is not equivalent to validation in a fully independent external cohort and does not eliminate the possibility of overfitting. Finally, the subset analyses examining post-surgical changes were limited by small sample size and should be regarded as preliminary.

The use of the SHUM framework was intended to optimize the combination of multiple biomarkers in the setting of ordered multicategory outcomes and correlated marker distributions. Because different statistical learning methods can yield different performance estimates, future work should evaluate the robustness of the identified signature across alternative modeling approaches, including penalized regression, random forest, and other classification frameworks. Such comparisons will help determine whether the observed performance is intrinsic to the EV biomarker signal or materially dependent on the chosen modeling strategy.

Overall, our findings identify a reproducible EV-associated protein signature linked to early-stage HGSOC within a retrospective case–control setting and support the EV compartment as a biologically informative source of circulating biomarkers. The results justify further testing in rigorously designed prospective studies that include clinically relevant benign comparators, symptomatic patients, high-risk women, and, ideally, pre-diagnostic longitudinal samples.

## 5. Conclusions

**Figure 4:**
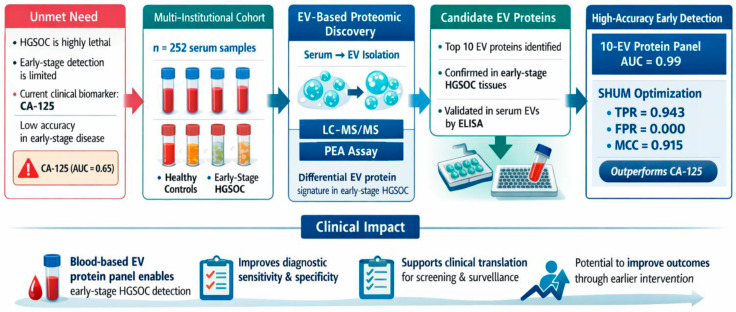
**Study workflow and diagnostic performance of the EV protein biomarker panel for early-stage HGSOC detection**. Serum EVs from a multi-institutional cohort (n = 252) were analyzed by LC–MS/MS and PEA for biomarker discovery, followed by ELISA validation. A 10-EV protein panel achieved high diagnostic accuracy for early-stage HGSOC (AUC = 0.99), outperforming CA-125 (AUC = 0.65). SHUM-based integration further enhanced classification performance, supporting clinical translation for early detection.

In summary, this study identified a panel of serum EV-associated proteins linked to early-stage HGSOC in a retrospective multi-institutional case–control cohort. Several candidate proteins were enriched in the EV fraction but not in whole serum, supporting the EV compartment as a potentially informative source of circulating tumor-associated biomarkers. A combined multi-protein model showed strong analytic discrimination between early-stage HGSOC and healthy controls within the study dataset; however, these findings should be interpreted as biomarker discovery results rather than evidence of established screening or diagnostic performance (Figure 4). External validation in clinically representative and prospectively collected cohorts will be essential to determine the generalizability and translational relevance of this EV-associated protein signature.

## 6. Future Directions

Future studies should validate these EV-associated protein candidates in larger, independent multi-institutional cohorts that include clinically relevant comparator groups, such as benign gynecologic conditions, inflammatory pelvic disease, adnexal masses, and nonmalignant symptomatic presentations. Prospective evaluation in high-risk populations and, where feasible, in pre-diagnostic longitudinal sample sets will be especially important to determine whether these biomarkers identify occult disease before clinical diagnosis or instead reflect specific HGSOC phenotypes detectable only after disease establishment. Additional studies should also examine the effects of pre-analytical variables, collection site, and alternative statistical modeling approaches on biomarker performance. Ultimately, these efforts will determine whether EV-associated protein signatures can be developed into clinically deployable assays for risk stratification, diagnostic triage, or earlier detection of HGSOC.

## Data Availability

The original contributions presented in this study are included in the article/Supplementary Materials. Further inquiries can be directed to the corresponding authors.

## Supplementary Materials

The following supporting information can be downloaded at https://www.mdpi.com/article/10.3390/cells15080706/s1: Table S1: Existing patient serum samples. Figure S1: EVs isolation and confirmation using different MFD chip and labs. Figure S2: Confirmation of EVs Surface Markers. Figure S3: Analysis of EVs Size and Concentration. Figure S4: IPA comparative core analysis reveals. Table S2; Comparison of the number of proteins identified based on spectral count. Figure S5: Confirmation of Candidate Proteins by IHC. Figure S6. SHUM Method. Figure S7: Shum Method. Figure S8: Comparison of the Area under the Curve.

## Abbreviations

AUC—Area under curve; CCNE1—Cyclin E1; CFH—Complement factor H; CP—Cisplatin; CV—Coefficient of variation; EHUM—Empirical estimate of HUM; EVs—Extracellular vesicles; FAS—Fatty acid synthase; FPR—False-positive rate; FT—Fallopian tube; HGSOC—High-grade serous ovarian cancer; HUM—Hyper-volume Under Manifolds; ISF—Image stream flow cytometry; LC-MS/MS—Liquid chromatography–mass spectrometry; LMM—Linear mixed-effects model; MCC—Mathew’s correlation coefficient; MFD—Microfluidics device; NID1—Nidogen1; NTA—Nanoparticle tracking analysis; OC—Ovarian cancer; PEA—Proximity extension assay; PZP—Pregnant zone protein; ROC—Receiver operating characteristic; SHUM—Smoothed empirical estimate of HUM; SPP24—Secreted phosphoprotein 24; STAT3—Signal transducer and activator of transcription 3; STIC—Serous tubal intraepithelial carcinoma; TEM—Transmission electron microscopy; TPR—True-positive rate; UT—Ultracentrifugation.
