# Supplementary material for: Serum Extracellular Vesicle Protein Signatures Associated with Early-Stage High-Grade Serous Ovarian Carcinoma
Source: Cells. 2026 Apr 16;15(8):706. doi: 10.3390/cells15080706 (PMC13114834; doi:10.3390/cells15080706)
Supplement: Supplementary file 1 [file cells-15-00706-s001.zip › cells-4233259 -Supp. Fig and Tables New.pdf]

Supplemental Table S1: Existing patient serum samples (early, late stage HGSOC and control (without cancer) used for this study from PI and collaborators institutions.

Sample source	Characteristic	Control	STAGE I/II	STAGE III/IV
**The Ohio State University**	**Number**	65	30	43
	**Mean of Age in years range**	56 (26-79)	61 (53-78)	60 (45-78)
	**Histology**	NA	HGSOC	HGSOC
**INOVA**	**Number**	15	21	40
	**Mean of Age in years range**	54 (30-76)	58 (41-84)	63 (40-83)
	**Histology**	NA	HGSOC	HGSOC
**Washington University**	**Number**	NA	14	NA
	**Mean of Age in years range**	NA	59 (40-72)	NA
	**Histology**	NA	HGSOC	NA
**Cedar's Sinai Cancer Institute**	**Number**	5	12	5
	**Mean of Age in years range**	59 (28-76)	62 (39-81)	64 (46-81)
	**Histology**	NA	HGSOC	HGSOC

Sup. Fig. 1

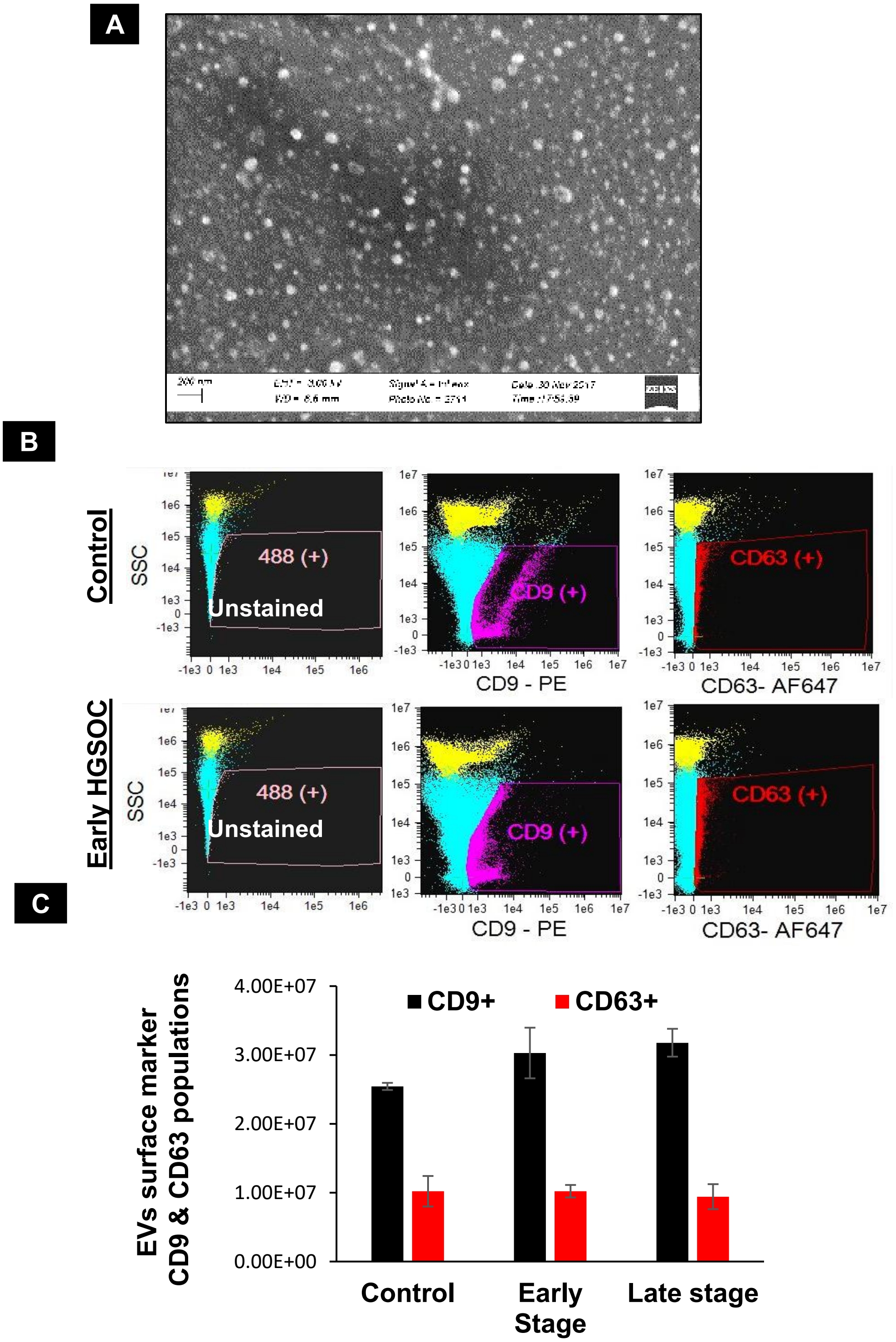

Sup. Figure S1. EVs isolation and confirmation using different MFD chip and labs. **A)** Morphological characterization and size measurement of EVs (indicated by red arrows) using Electron Microscopy (EM). **B &C**: Representative Image stream analysis for EV surface markers- CD9 and CD63 performed on Control, Early and Late Stage HGSOC patient samples.

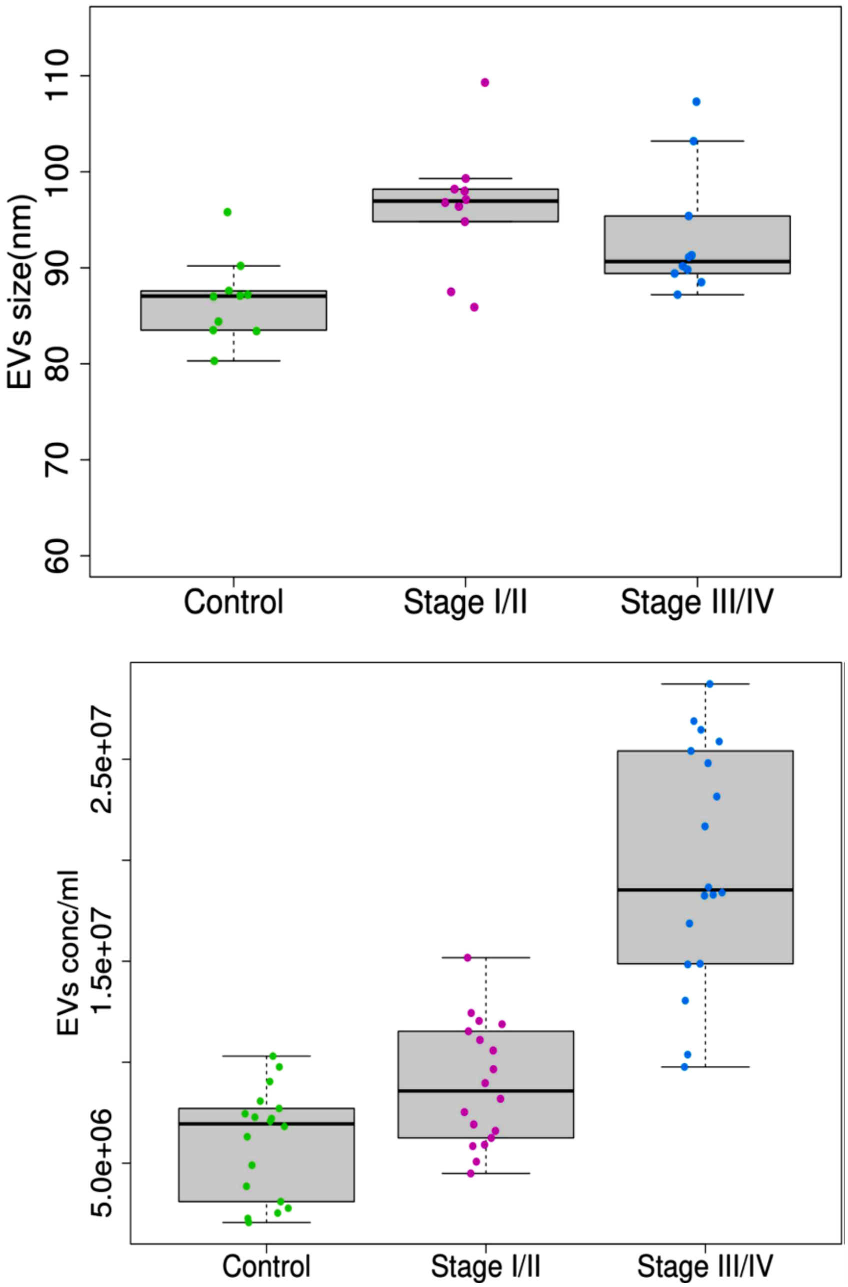

Sup. Figure S2. Confirmation of EVs Surface Markers. Confirmation of the presence of specific surface markers CD9 and CD63 on EVs populations using flow cytometry in serum samples from different HGSOC patients.

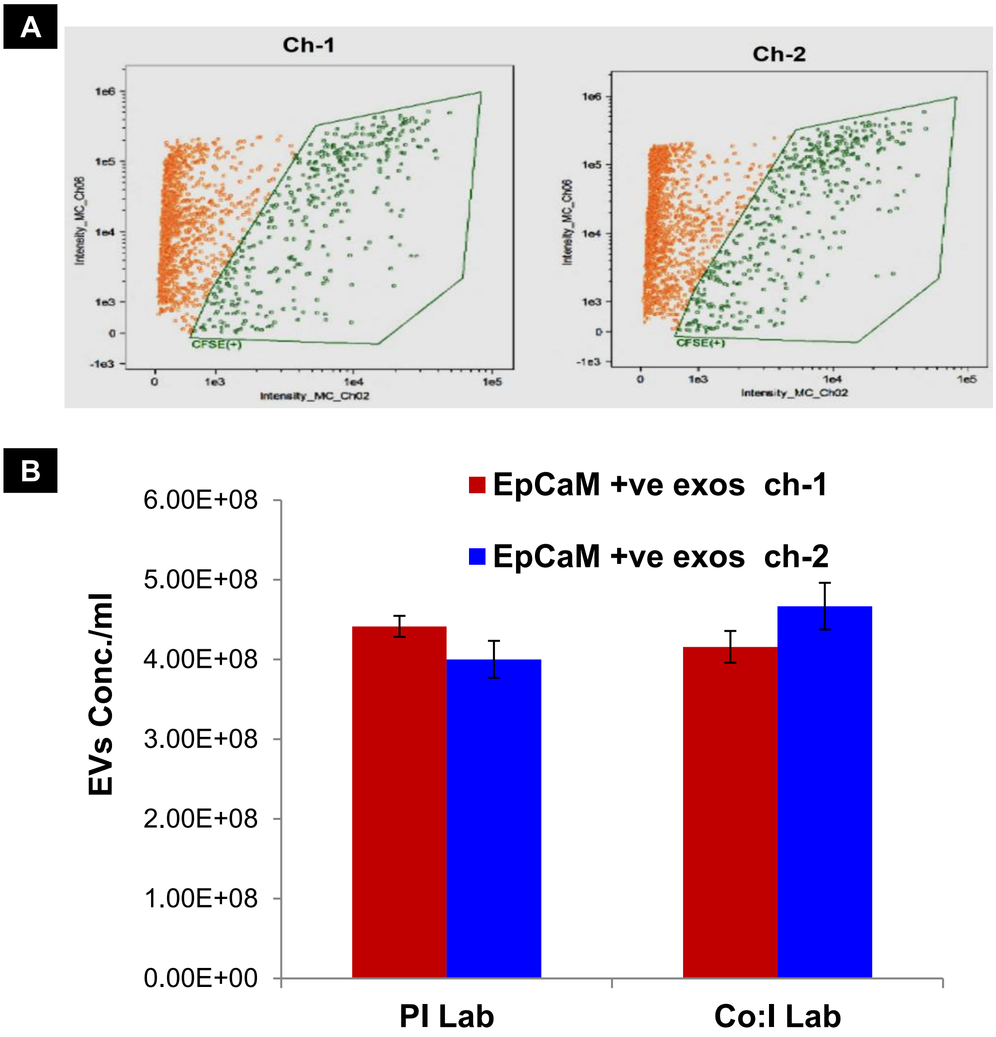

Sup. Figure S3. A) Exosome particle count (green) measured by Image stream flow cytometer to show the technical reproducibility of different MFD channels (Ch-1 or Ch-2) using same patient sample. B**)** Comparative Nanoparticle tracking analysis (NTA) for total EpCAM positive exosome tested in two different labs (PI and Co-I) with our MFD channels shows good reproducibility and reliability.

IPA core analysis on the top canonical pathways & diseases and functions in our data set

A

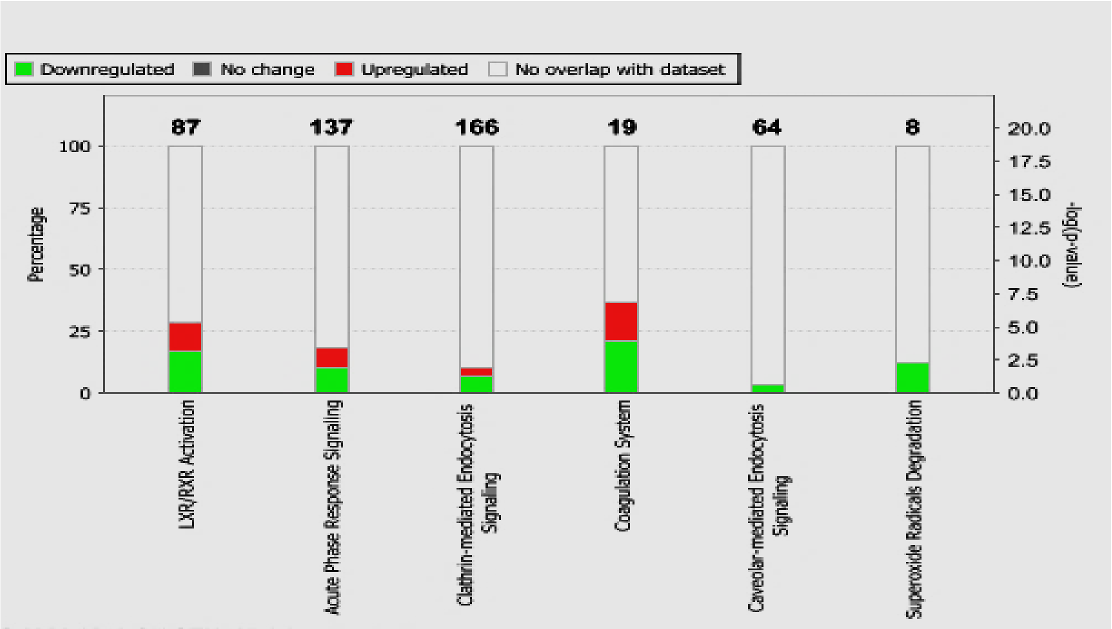

B

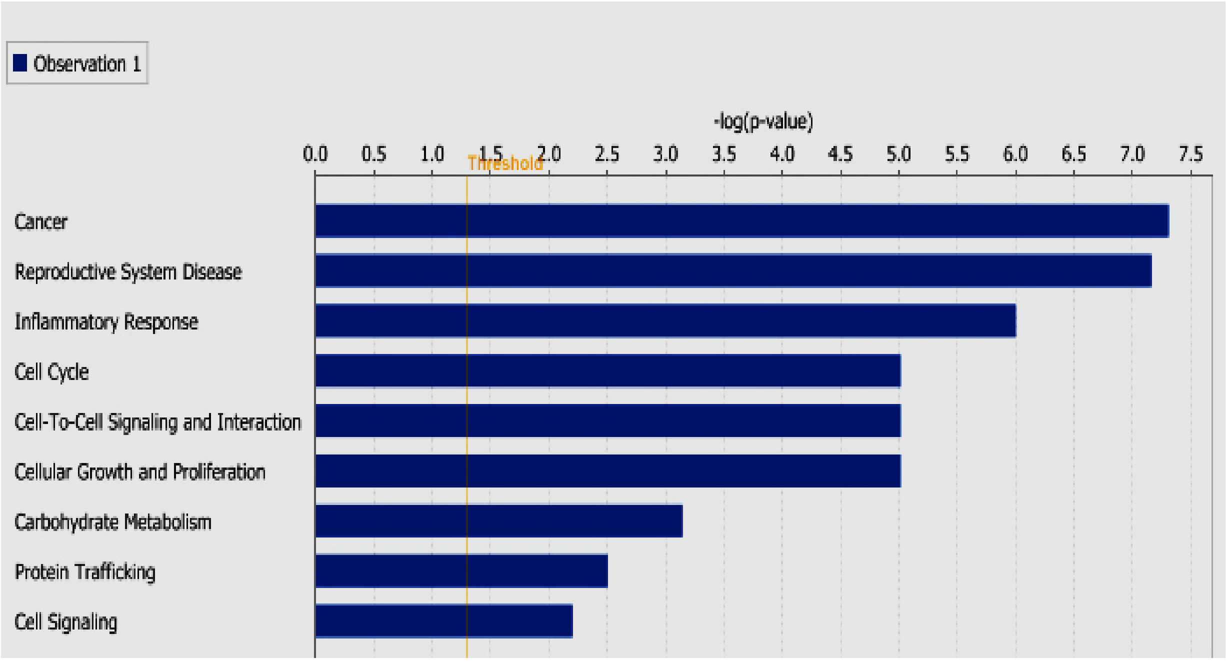

Sup Figure S4: IPA comparative core analysis reveals **(A)** the top canonical pathways and the percentage of upregulated and downregulated proteins identified in our dataset expressed as log (P value) in Early HGSOC stage(I&II) Vs Control healthy patient samples. **(B)** The top disease and functions associated with the proteins identified in our dataset are expressed as log (P value) above a significance threshold based on Fisher exact test ($P < 0.05$).

Sup. Table S2. Comparison of the number of proteins identified based on spectral count(SC) and abundance (Abun) after filtering lowly expressed proteins, showing significant proteins at p-value and at q-value. The tims TOF identified more proteins overall based on the Abundance and the exclusion of the "outliers" helped the statistics in DE analysis at q-value.

Analysis method	Protein ID's	Proteins after filtering	Sig. protein ID's at pValue	Sig. protein ID's at qValue
tims_ SC_ All	533	250	24	1
tims_ SC_ Sel	533	245	38	0
tims_ Abun_All	426	288	74	16
tims_Abun_Sel	426	292	92	73

Sup. Fig. 5

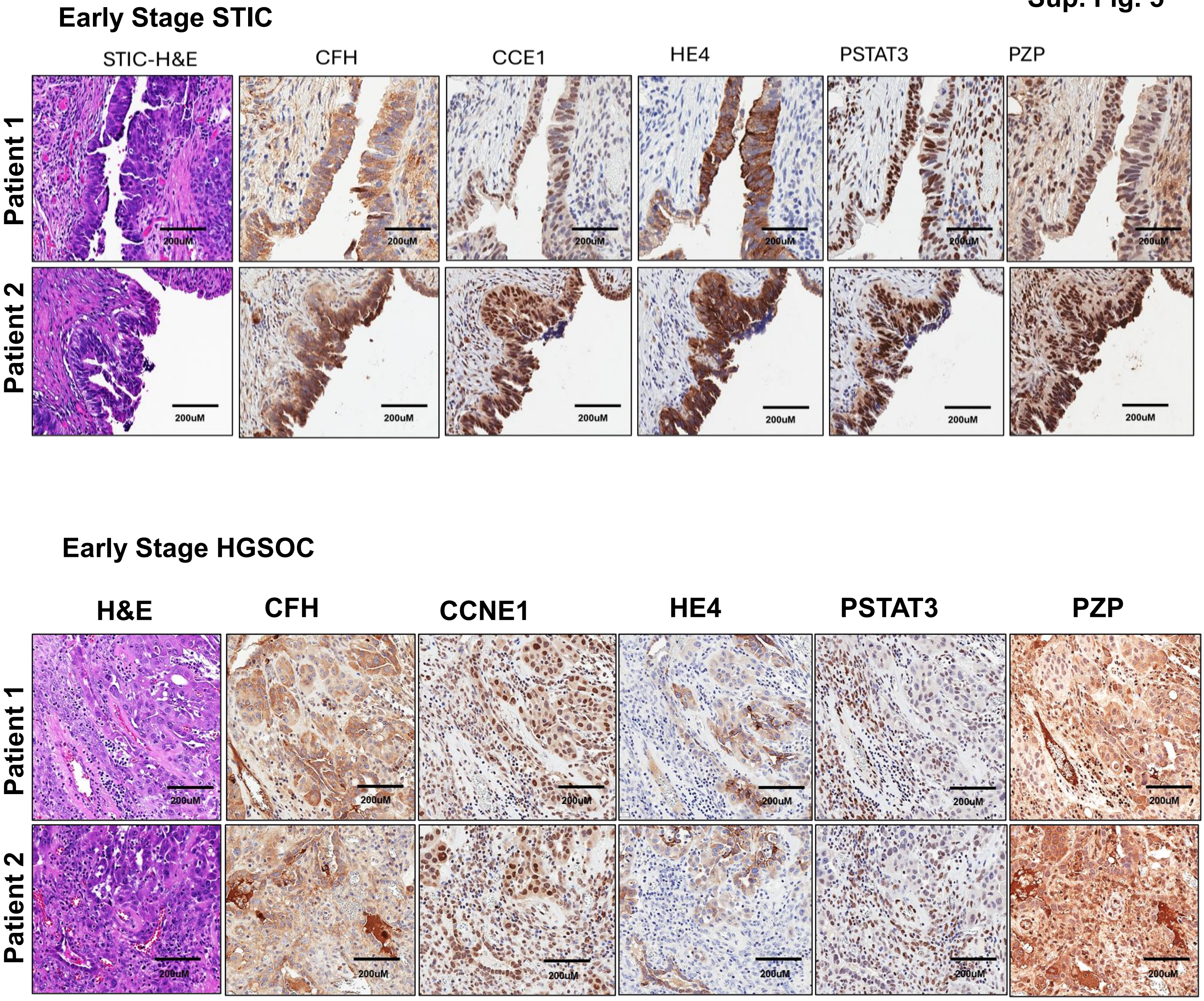

Sup. Figure S5. Confirmation of Candidate Proteins (CFH, CCNE1, pSTAT3 and PZP with known biomarker of HE4 in STIC and Early-stage HGOSC patient tissues using IHC.

Sup. Fig. 6

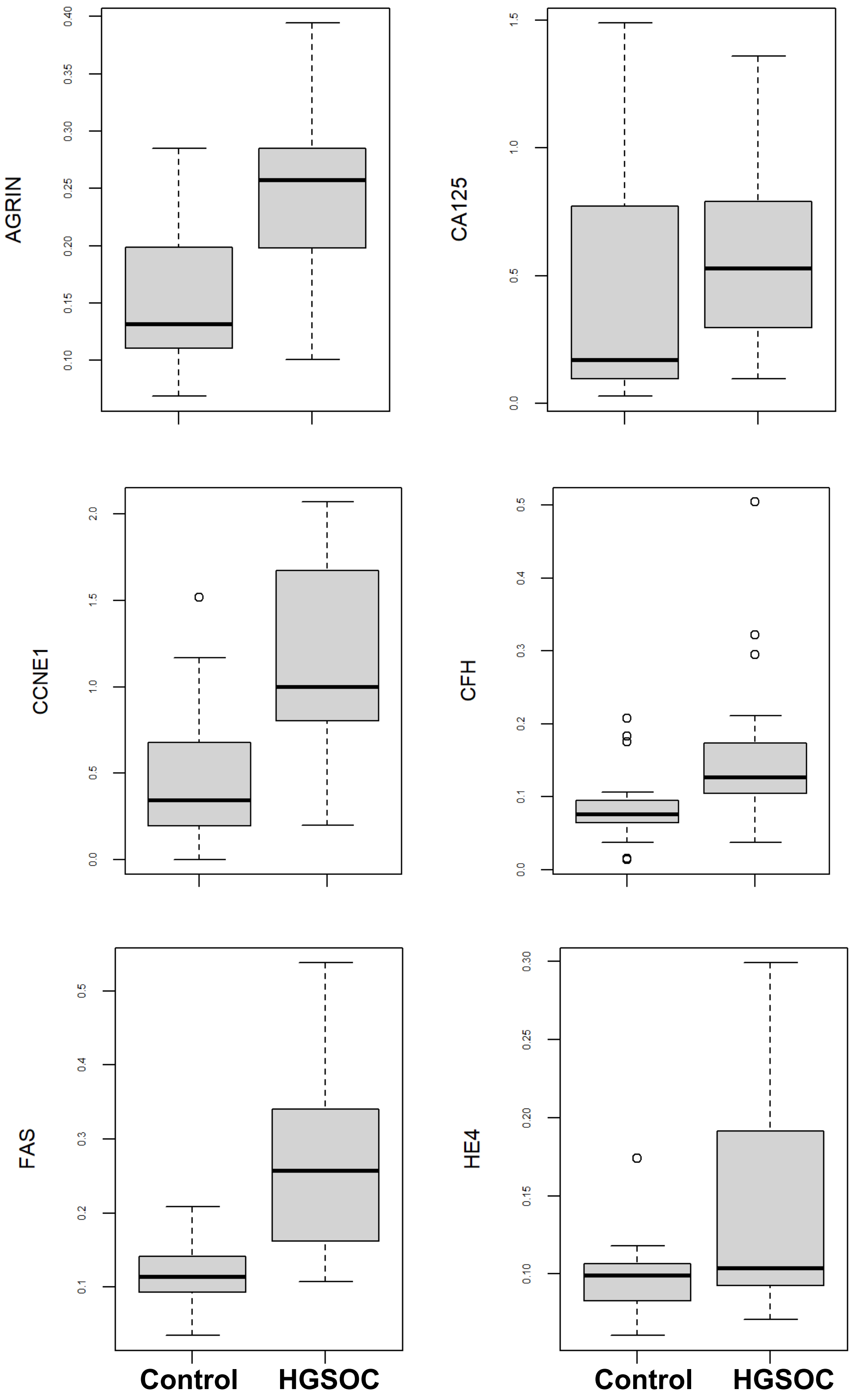

Sup. Figure S6. Data from 12 protein biomarkers (AGRIN, CA125(MUC16), CCNE1, CFH, FAS, HE4, IL-6, NID1, PDL1, PZP, SPP24, STAT3) were used to classify patient groups (Control and HGSOC Early-stage). The optimization algorithm proposed by Das et al. (2022) was used to maximize a smoothed estimate of hyper-volume under manifolds (SHUM).

Sup. Fig. 7

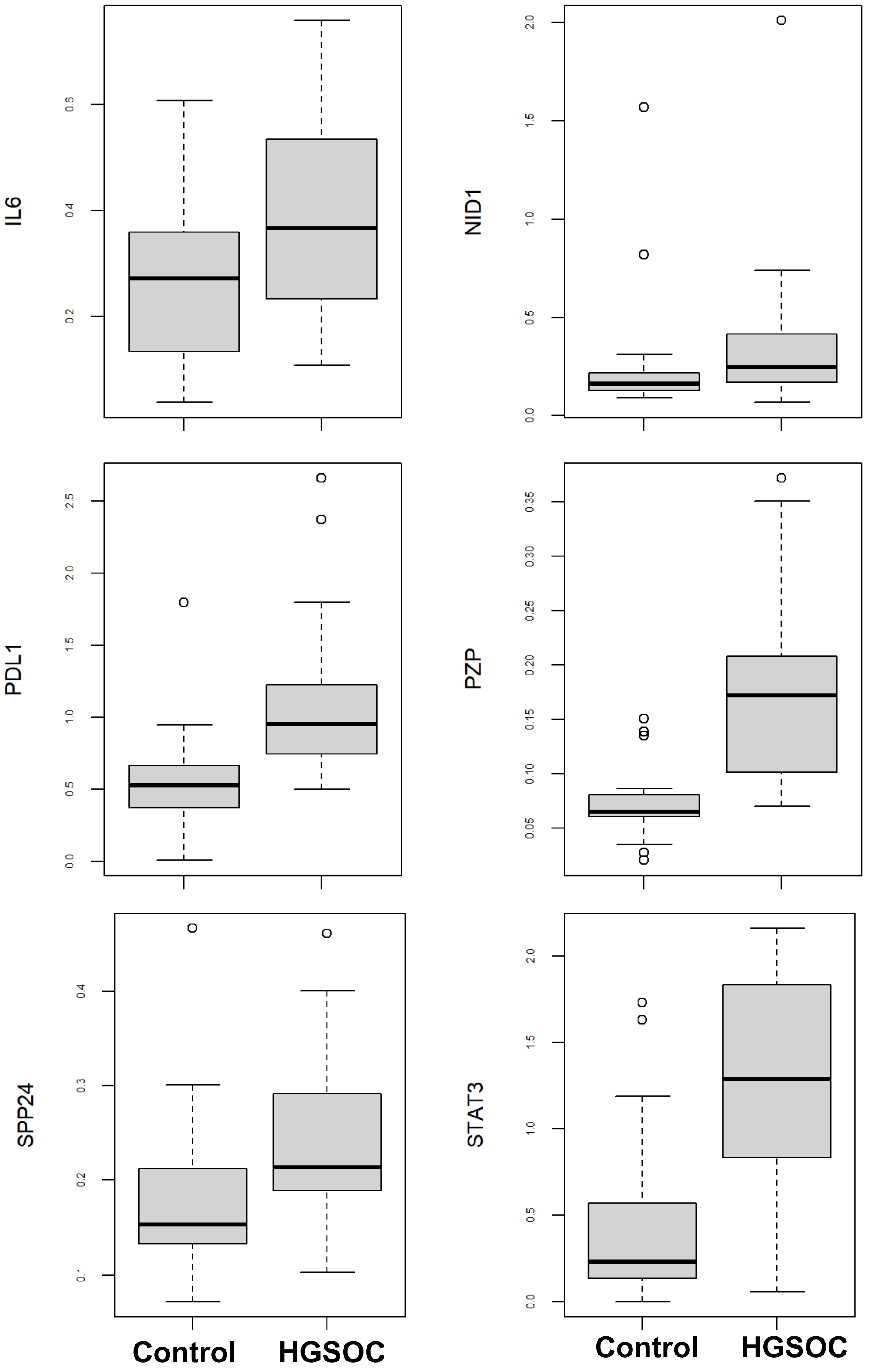

Sup. Figure S7. Data from 12 protein biomarkers (AGRIN, CA125(MUC16), CCNE1, CFH, FAS, HE4, IL-6, NID1, PDL1, PZP, SPP24, STAT3) were used to classify patient groups (Control and HGSOC Early-stage). The optimization algorithm proposed by Das et al. (2022) was used to maximize a smoothed estimate of hyper-volume under manifolds (SHUM).

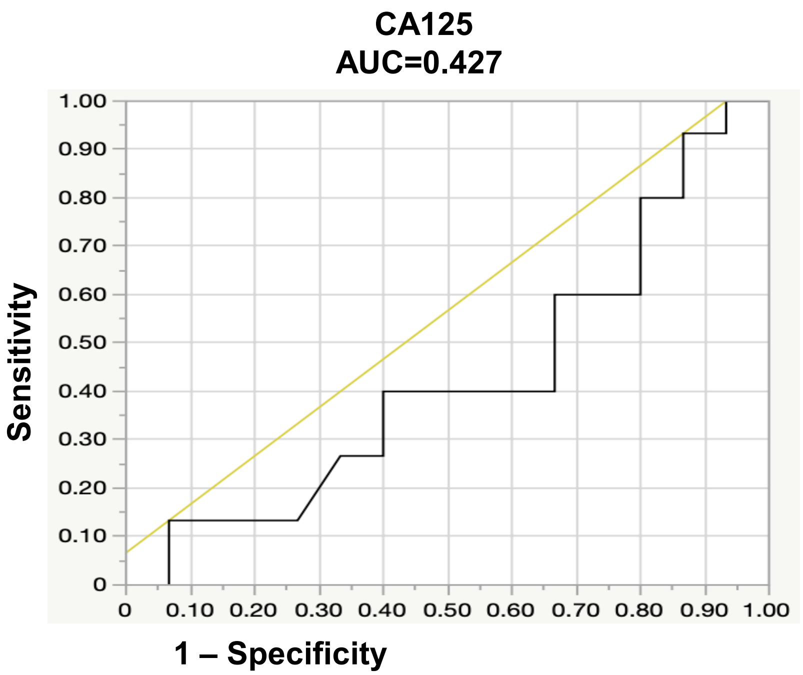

Sup. Figure S8. Comparison of the Area under the Curve (AUC) in the validation cohort, assessing the predictive performance of CA125 expression as a biomarker in early-stage High-Grade Serous Ovarian Cancer (HGSOC) samples versus control serum samples.
